# Supplementary material for: Impact of climate change on millet yield under different fertilization levels in three agroecological zones of Niger Republic
Source: PLoS One. 2025 Oct 9;20(10):e0333963. doi: 10.1371/journal.pone.0333963 (PMC12510525; doi:10.1371/journal.pone.0333963)
Supplement: S1 File — (DOCX) [file pone.0333963.s001.docx]

Inclusivity in global research

PLOS’ policy on inclusivity in global research aims to improve transparency in the reporting of research performed outside of researchers’ own country or community and ensures that PLOS publications reporting global research adhere to high standards for research ethics and authorship. Authors of relevant research articles may be asked to complete the questionnaire below, which outlines ethical, cultural, and scientific considerations specific to inclusivity in global research. This questionnaire may be requested when researchers have travelled to a different country to conduct research, if research uses samples collected in another country, research with Indigenous populations or their lands, or if research is on cultural artefacts. Researchers travelling to another country solely to use laboratory equipment will not normally be required to complete the questionnaire. However, the questionnaire can be requested at the journal’s discretion for any submission – if you have been requested to complete this questionnaire by the PLOS journal you submitted to, please do so.

Please complete the questionnaire below and include this as a Supporting Information file with your manuscript. Note that if your paper is accepted for publication, this checklist will be published with your article in the supporting information files. Please ensure that you reference the checklist in the main body of your manuscript. We suggest adding a subsection ‘Inclusivity in global research’ to your Methods section and adding the following sentence: “Additional information regarding the ethical, cultural, and scientific considerations specific to inclusivity in global research is included in the Supporting Information (SX Checklist)”

The questions have been designed to be applicable to a wide range of study types, and there are subsections for both human subjects research and non-human subjects research. If any of the questions are not relevant to your research please mark them as “N/A” as appropriate.

**Ethical considerations, permits and authorship**

*This section is applicable to all research types.*

Provide details as to who granted permissions and/or consent for the study to take place in the Methods section of your manuscript. This should include the names of **all** ethics boards, governmental organizations, community leaders or other bodies that provided approval for the study. If individuals provided approval refer to these people by their role or title but do not list their name(s).

This study was led by the Institut National de la Recherche Agronomique du Niger, is a government agency mandated with undertakeing and coordinating research related to agriculture in Niger. It is written in the constitution of the country. This research was therefore a product of the mandate of the Institut National de la Recherche Agronomique du Niger. The same institution therefore provided the approval.

**Reported on page number 5: The research was approved by these organisations below:**

**Institut National de la Recherche Agronomique du Niger, BP : 429 Niamey, Niger**

**WASCAL, Competence Center Blvd Moammar El-Khadafi, BP: 9507, Ouagadougou, Burkina Faso**

**International Institute of Tropical Agriculture, IITA-Kenya, of ILRI, PO Box 307009-00100, Nairobi, Kenya**

**University of Twente, Department of Natural Ressources, Netherlands**

**Université du Sine Saloum El Hadj Ibrahima Niass 55 Kaolack, Senegal**

**Federal College of Horticulture Dadin Kowa, Gombe State, Nigeria**

**Department of Agronomy, Bayero University, Kano, Nigeria**

**Climate Hazards Center (CHC), University of California, Santa Barbara, United States.**

**These organisations are mandated by their host countries to undertake this research. There is need for ethical clearance only if there is direct contact with human beings e.g. through a survey. If the study does not involve physical contact with human beings, there is no need to seek an advanced ethical approval.**

If there were any deviations from the study protocol after approval was obtained please provide details of these changes in the Methods section of your manuscript.
Did this study involve local collaborators that are residents of the country where the research was conducted or members of the community studied? If you do not have any authors from said communities, please provide an explanation for this below. **This study did not involve local collaborators that are residents of the country where the research was conducted or members of the community studied.**

Reported on page number:

**There were no deviations.**

Everyone listed as an author should meet PLOS’ criteria for authorship and all individuals who meet these criteria should be included in the author byline, rather than the acknowledgements. For further information please see the journal’s Authorship Policy.

**Human subjects research (e.g. health research, medical research, cross-cultural psychology)**

Did you obtain written informed consent from a representative of the local community or region before the research took place? How did you establish who speaks for the community? Details of written informed consent obtained from study participants should be reported separately in the Methods section of your manuscript.

**The organisations have mandate from the government to undertake this research. This government mandate also covers for local communities.**

How did members of the local community provide input on the aims of the research investigation, its methodology, and its anticipated outcome(s)?

**The study used literature from the region and country. This provided more context which matches with that from the local community.**

When engaging with the local community, how did you ensure that the informed consent documents and other materials could be understood by local stakeholders? **This studies did not involve engagements with the local communities.**

**This study did not involve engagement with the local community as it is crop simulations modelling**

Will the findings of the research be made available in an understandable format to stakeholders in the community where the study was conducted (e.g. via a presentation, summary report, copies of publications, etc.)? Please provide details of how this will be achieved.

The research from this study is being prepared for presentation to the government and the ministry of agriculture and environment. The material is also being formatted to a policy brief so that policy makers are aware of the impacts of climate on millet. The copies of the publications will be given to the technical heads of the ministries and local universities.

**Non-human subjects research using specimens/ animals collected as part of the study, or those housed in archival collections. Examples include archaeology, paleontology, botany and zoology.**

Did the permission you obtained from a local authority to perform the study include an agreement on access to outputs and benefit sharing? This may include procedures to enable fair distribution of the benefits and resources arising from the research performed. Please include any details of Prior Informed Consent and Benefit Sharing Agreements obtained. These may be required by field-specific regulations, for example the Convention on Biological Diversity (CBD) and the associated Nagoya Protocol.

**No Benefit Sharing Agreements obtained or agreed to.The country government – institution agreement was to underake research which is beneficial to the country. This research therefore meets that criteria.**

If the material used in your study was imported, please A) provide the year it was imported and B) indicate whether permits were obtained to import/export the materials used, C) provide details of any permits obtained. If this information is not available, please indicate this.

**No material was imported.**

If you used archival specimens, please state how the material used in your study was acquired by the institute it is held in and provide details of any permits obtained for the original excavations/ sample collection. If this information is not available, please indicate this.

**No archival specimens were used.**

How was the potential cultural significance of the materials collected in your study to local communities considered in your research design? Were Indigenous peoples and/or local researchers and institutions involved with archaeological excavations / collection of specimens? If so, please provide a description of their involvement.

**No materials were collected. In addition, no archeological excavations were undertaken or specimens collected.**

If your manuscript includes photographs of human remains please indicate whether authors obtained permission from descendants or affiliated cultural communities to do so.

**No photographs of human remains were collected in this study and or included in this manuscript.**
