# Supplementary material for: Impact of climate change on millet yield under different fertilization levels in three agroecological zones of Niger Republic
Source: PLoS One. 2025 Oct 9;20(10):e0333963. doi: 10.1371/journal.pone.0333963 (PMC12510525; doi:10.1371/journal.pone.0333963)
Supplement: Attachment — Submitted filename: Response to Reviewers.docx [file pone.0333963.s003.docx]

**Reviewer comments 1:**

When submitting your revision, we need you to address these additional requirements***.***

**Response:**
Thank you for your comment. We have carefully reviewed and addressed all the additional requirements in the revised manuscript.

**2 Reviewer Comment:**

In your Methods section, please provide additional information regarding the permits you obtained for the work. Please ensure you have included the full name of the authority that approved the field site access and, if no permits were required, a brief statement explaining why**.**

**Author Response:**

No specific permits were required for this study, as the data were collected from privately managed land under my direct supervision and ownership. I am solely responsible for the data collection and access, and no additional field site approvals were necessary.

**3. Code Sharing**

**Editorial Note:**

Ensure code is shared according to PLOS ONE guidelines for reproducibility and reuse.

**Response:**
All author-generated code has been shared in accordance with PLOS ONE guidelines. The code repository is publicly available at: <https://github.com/smkuhlani/ClimateChangeImpacts_Millet_Niger>

**4 Editorial Note:**

Please include your tables as part of your main manuscript and remove the individual files. Supplementary tables should remain uploaded as separate supporting information files.

**Response:**

This has been addressed. All main tables have been incorporated into the manuscript

**5 Editorial Note:**

Please provide a complete Data Availability Statement, including access details or a reason for any restrictions. If all data are in the manuscript, state: "All data are in the manuscript

**Response:**

**Data Availability Statement**

**Data Availability**

All data generated or analyzed during this study are available at the Figshare repository, accessible via the following DOI link: [**https://doi.org/10.6084/m9.figshare.29951645**](https://doi.org/10.6084/m9.figshare.29951645)

**7 Editorial Note:**

Please amend either the title on the online submission form or the manuscript so that they are identical.

**Response:**

This has been handled. The title in the manuscript and the online submission form now match exactly.

**8 Editorial Note:**

Figure 1 contains map images that may be copyrighted. PLOS cannot publish maps created using proprietary data such as Google Maps.

**Response:**

Thank you for the note. Although the map was created by the author. To ensure full compliance with PLOS policies, the map has been removed from the manuscript.

**9 Editorial Note:**

Please review reviewer-suggested citations and include them if relevant.

**Response:**

The reviewer did not recommend citing any specific previously published works. Therefore, no additional citations were added.

**Reviewer #1**

**Reviewer Comment:**

Please briefly mention the calibration procedure (location and time) and specify how many and which GCMs were used in the abstract.

**Author Response:**

This information has been added to the abstract, including details on the calibration procedure (location and time) and the number and names of the GCMs used

**Reviewer Comment:**

The manuscript lacks line numbers, making it difficult to provide detailed comments.

**Author Response:**

Line numbers have been added to the revised manuscript to facilitate review.

**Reviewer Comment:**

Please start the introduction by stating where the Sahel region is located and why it is favorable for millet cultivation (e.g., spring temperatures, rainfall). Also, clarify whether the 62% figure refers to total millet production or total cereal production in the region.

**Author Response:**

This has been addressed. The introduction now includes a brief description of the Sahel region’s location and its suitability for millet cultivation. Additionally, the 62% figure has been clarified to refer to national millet production

**Reviewer Comment:**

Please include a table summarizing model parameters, especially if multiple cultivars were used, and provide relevant cultivar information. Replace “evaluation” with “validation” throughout. Use the full model name-DSSAT-CERES-Millet—consistently instead of just “DSSAT.”

**Author Response:**

This has been taken care of. A table summarizing model parameters and cultivar details has been added. All instances of “evaluation” have been replaced with “validation,” and the full model name DSSAT-CERES-Millet is now used consistently throughout the manuscript.

**Reviewer Comment:**

Please discuss study limitations more explicitly and suggest future improvements. Strengthen novelty by assessing nitrogen stress under low-fertility conditions, and discuss implications for fertilizer application due to early flowering and maturity. Consider citing relevant previous studies.

**Author Response:**

This has been updated. The discussion now explicitly addresses study limitations, future improvements, and the impact of nitrogen stress under low-fertility scenarios. Implications for fertilizer strategies related to phenological shifts have also been added, supported by relevant literature

**Reviewer Comment:**

Please expand the conclusion with more actionable insights, such as shifting sowing windows, breeding suitable ideotypes, and aligning fertilizer management with changing crop phenology.

**Author Response:**

The conclusion has been expanded to include actionable insights on adjusting sowing windows, breeding appropriate ideotypes, and aligning fertilizer management with evolving crop phenology

**Reviewer Comment:**

Please add standard deviation or error bars to figures and conduct ANOVA for significant differences among GCM outputs. For Figure 9, omit biomass-related outputs since biomass data were not used for calibration.

**Author Response:**

We acknowledge the suggestion to include standard deviations and conduct ANOVA. However, each data point represents a single value from one model–scenario–time horizon combination, with no replicates, so variability measures and ANOVA were not possible. As recommended, biomass-related outputs have been omitted from Figure 9 since biomass data were not used in model calibration.

**Reviewer #2**

**Reviewer Comment:** According to the submitted manuscript standards of the journal, you would need to number the lines all over the manuscript with a continuous numbering.

**Response:** Thank you for the helpful suggestion. We have revised the manuscript to include continuous line numbering throughout the entire document, in accordance with the journal's submission guidelines.

**Reviewer Comment:**

In the abstract, in line 6, you need to substitute the word *"reduced"* with *"shortened"*. In line no. 9, you need to put the word *"scenarios"* instead of *"models"*. In line no. 11, you need to correct the phrase to *"Climate Change's threat"*.

**Response:**

Thank you for your constructive suggestions. We have revised the abstract as follows:

- The word **"reduced"** in line 6 has been replaced with **"shortened"**.
- The word **"models"** in line 9 has been changed to **"scenarios"**.
- The phrase in line 11 has been corrected to **"Climate Change's threat"**.

**Reviewer Comment:**

In the conclusions, in line no. 1, you need to correct the phrase to *"has been proven"*. In line no. 4, you need to correct the phrase to *"on the study"*.

**Response:**

Thank you for pointing out these issues. We have made the necessary corrections in the conclusion section:

- The phrase in line 1 has been corrected to **"has been proven"**.
- The phrase in line 4 has been revised to **"on the study"**
