# Supplementary material for: Impact of climate change on millet yield under different fertilization levels in three agroecological zones of Niger Republic
Source: PLoS One. 2025 Oct 9;20(10):e0333963. doi: 10.1371/journal.pone.0333963 (PMC12510525; doi:10.1371/journal.pone.0333963)
Supplement: Attachment — Submitted filename: Response_to_Reviewers_auresp_2.docx [file pone.0333963.s004.docx]

**Editorial Comment:**

*If the reviewer comments include a recommendation to cite specific previously published works, please review and evaluate these publications to determine whether they are relevant and should be cited. There is no requirement to cite these works unless the editor has indicated otherwise.*

Response:

We confirm that no specific previously published works were suggested by the reviewers. Accordingly, no additional citations were required. We have carefully reviewed the comments and made relevant additions and clarifications where appropriate**.**

**Editorial Comment:**
*Please review your reference list to ensure that it is complete and correct. If you have cited papers that have been retracted, please include the rationale for doing so in the manuscript text, or remove these references and replace them with relevant current references. Any changes to the reference list should be mentioned in the rebuttal letter that accompanies your revised manuscript. If you need to cite a retracted article, indicate the article’s retracted status in the References list and also include a citation and full reference for the retraction notice.*

**Author Response:**
We have carefully reviewed the entire reference list for completeness and accuracy. No retracted papers have been cited in the manuscript. Minor formatting and citation style corrections have been made in accordance with PLOS ONE referencing guidelines. Additionally, a few references have been updated or replaced with more recent and relevant publications where appropriate. All changes made to the reference list are reflected in the revised manuscript.

**Reviewer #2 Comment**

*“The abstract is reasonable. However, please briefly mention the calibration procedure (location and time), and specify how many and which GCMs were used.”*

Response:

We thank the editor for the helpful suggestion. We have revised the abstract to briefly describe the calibration procedure, including the locations (Goungoubon and Fandou) and the years (2021 and 2022) during which field data were collected. We have also specified the number and names of the five Global Circulation Models (GCMs) used in the study. The revised section in the abstract now reads:

*“The CERES-Millet model was calibrated using field data collected during the 2021 and 2022 rainy seasons at two sites in Niger: Goungoubon (2021 and 2022) and Fandou (2022). Trials were conducted near irrigation facilities to ensure optimal moisture conditions. Calibration involved iterative adjustment of genetic coefficients based on observed phenology and grain yield. Future climate impacts were assessed using five GCMs with varying climate sensitivity: IPSL-CM6A-LR, MPI-ESM1-2-HR, GFDL-ESM4, MRI-ESM2-0, and UKESM1-0-LL.”*

***Reviewer Comment:***

*Please start the introduction by stating where the Sahel region is located and why it is favorable for millet cultivation (e.g., spring temperatures, rainfall). Also, clarify whether the 62% figure refers to total millet production or total cereal production in the region.*

***Author Response:***

*This has been addressed. The introduction now includes a brief description of the Sahel region’s location and its suitability for millet cultivation. Additionally, the 62% figure has been clarified to refer to national millet production*

**Comment:**

*“It was difficult to note down detailed comments as the manuscript lacks line numbers—please include them in future versions.”*

**Response:**

Thank you for the observation. Line numbers have now been added to the revised manuscript to facilitate easier review.

**Comment:**

*“Please include a table summarising model parameters, especially if multiple cultivars were used. Provide relevant cultivar information.”*

**Response:**

In response, we have added **Table 2** to the revised manuscript, which summarizes the genetic coefficients used in the CERES-Millet model for the millet cultivars evaluated. The table includes cultivar names, parameter values, and descriptions of each parameter to ensure clarity and reproducibility.

**Comment:**

*Please replace “evaluation” with “validation” throughout the manuscript. Also, instead of just writing “DSSAT,” use the full model name DSSAT-CERES-Millet consistently.*

**Response:**

We thank the editor for this helpful clarification. We have revised the manuscript accordingly by replacing all instances of “evaluation” with “validation” and have consistently used the full model name **“DSSAT-CERES-Millet”** throughout the text.

**Comment:**

Please discuss study limitations more explicitly and suggest future improvements. Strengthen novelty by assessing nitrogen stress under low-fertility conditions, and discuss implications for fertilizer application due to early flowering and maturity. Consider citing relevant previous studies.

**Response:**

This has been updated. The discussion now explicitly addresses study limitations, future improvements, and the impact of nitrogen stress under low-fertility scenarios. Implications for fertilizer strategies related to phenological shifts have also been added, supported by relevant literature

**Comment:**

Please expand the conclusion with more actionable insights, such as shifting sowing windows, breeding suitable ideotypes, and aligning fertilizer management with changing crop phenology.

**Response:**

The conclusion has been expanded to include actionable insights on adjusting sowing windows, breeding appropriate ideotypes, and aligning fertilizer management with evolving crop phenology

**Reviewer Comment:**

Please add standard deviation or error bars to figures and conduct ANOVA for significant differences among GCM outputs. For Figure 9, omit biomass-related outputs since biomass data were not used for calibration.

**Author Response:**

We acknowledge the suggestion to include standard deviations and conduct ANOVA. However, each data point represents a single value from one model–scenario–time horizon combination, with no replicates, so variability measures and ANOVA were not possible. As recommended, biomass-related outputs have been omitted from Figure 9 since biomass data were not used in model calibration

**Reviewer #2 Comment:**

*According to the submitted manuscript standards of the journal, you would need to number the lines all over the manuscript with a continuous numbering. This would make our job as reviewers.*

**Response:**

We sincerely thank the reviewer for the positive feedback and kind words regarding the value and relevance of our study. In response to the comment, we have revised the manuscript to include **continuous line numbering throughout**, in accordance with the journal’s formatting guidelines.

**Reviewer #2 Comment:**

*In the abstract, in line 6, substitute the word “reduced” with “shortened.” In line 9, replace “models” with “scenarios.” In line 11, correct the phrase to “Climate Change’s threat.”*

**Response:**

Thank you for the helpful suggestions. We have made the requested changes in the abstract. Due to the addition of line numbers, the changes now appear as follows: the word “reduced” was replaced by “shortened” on line 33, “models” was replaced by “scenarios” also on line 33, and the phrase was corrected to “Climate Change’s threat” on line 38.

**Reviewer #2 Comment:**

*In the conclusions, in line no. 1, you need to correct the phrase to “has been proven.” In line no. 4, you need to correct the phrase to “on the study.”*

**Response:**

We thank the reviewer for these helpful suggestions. In the revised manuscript, the phrase was updated to **“has demonstrated strong effectiveness”** (now on line **572**) in place of “has been proven,” and the phrase **“on the study”** has also been updated as recommended
